# ISSR markers show differentiation among Italian populations of *Asparagus acutifolius *L

**DOI:** 10.1186/1471-2156-6-17

**Published:** 2005-03-18

**Authors:** Maria Sica, Graziella Gamba, Stefania Montieri, Luciano Gaudio, Serena Aceto

**Affiliations:** 1 Dipartimento di Genetica, Biologia generale e molecolare, Università degli Studi di Napoli Federico II, via Mezzocannone 8, 80134 Napoli, Italy

## Abstract

**Background:**

*Asparagus acutifolius *L. is a dioecious and native plant species, widely distributed in the Mediterranean Basin. It is known for its fine flavour and could represent an important resource for cultivation programs in desert areas. Few molecular studies have been performed on this species. In the present paper, the ISSR technique was employed to study genetic diversity in Italian *A. acutifolius*.

**Results:**

Twenty-three primers produced a total of 228 polymorphic fragments used to evaluate genetic variation. F_ST _(0.4561) and Theta B (0.4776) values indicate a wide genetic variation among the samples examined. The distance UPGMA tree grouped together the genotypes strictly according to their geographical origin, showing that each sample is genetically structured and can be considered a distinct population. AMOVA analysis further confirmed genetic structuring of the populations. Population-specific fragments were also detected.

**Conclusion:**

The results suggest that ISSR markers are useful in distinguishing the populations of *A. acutifolius *according to geographical origin, and confirm the importance of genetic studies for designing germplasm conservation strategies.

## Background

The availability of a variety of DNA markers, such as restriction fragment length polymorphism (RFLP), amplified fragment length polymorphism (AFLP), random amplified polymorphic DNA (RAPD), simple sequence repeat (SSR) and intersimple sequence repeat (ISSR) has enabled researchers to investigate genetic diversity among various plant species across natural populations [1-5]. Among these, PCR-based techniques of random multilocus analysis (RAPD, AFLP, ISSR) have been successfully used in genotyping, genome mapping and phylogenetic studies in horticultural crops such as strawberry [6], soybean [7], and potato [8].

Local populations of traditional cultivars provide a valuable resource for plant breeding as well as for the preservation of genetic diversity [9]. The exploration, evaluation, and conservation *in situ *and *ex situ *of genetic diversity in natural populations is imperative to guarantee sustainable development [10].

*Asparagus acutifolius *L. (Liliaceae) is a native, perennial plant species widely distributed throughout the Mediterranean areas, whose flowers are classified as dioecious and are mainly bee-pollinated; it generally does not reproduce by self-pollination. It grows in bushy and semi-dry places, sunny or semi-shade, mainly on limestone.

This species is known for its strong taste compared to the cultivated *A. officinalis *and does not require rich soils for cultivation; for these reasons, it could be an economically important resource for the recovery of arid rural areas where controlled introduced programs could be achieved.

To date, there is little information available on the genetic variability of this species. At present, the most widely studied species is *A. officinalis*, for which many molecular markers have been characterized (RAPD, RFLP, AFLP) [11]. The few molecular data regarding *A. acutifolius *are drawn from RAPD analyses [12] and the identification of microsatellite loci [13]. The ISSR technique is similar to that for RAPD, except that ISSR primers consist of a di- or trinucleotide simple sequence repeat with a 5' or 3' anchoring sequence of 1–3 nucleotides. Compared with RAPD primers, the ISSR primers sequence is usually larger, allowing for a higher primer annealing temperature which results in greater band reproducibiliy than RAPD markers [14]. They have been successfully used to assess genetic variation in plants such as citrus [15], *Viola pubescens *[14], potato [8], and *Oryza *[16].

In this study we used ISSR markers to analyse the genetic diversity of Italian *A. acutifolius *collecting samples in eight different scattered rural areas: six continental and one each from the Italian islands of Sardinia and Sicily.

## Results

**Figure 1:**
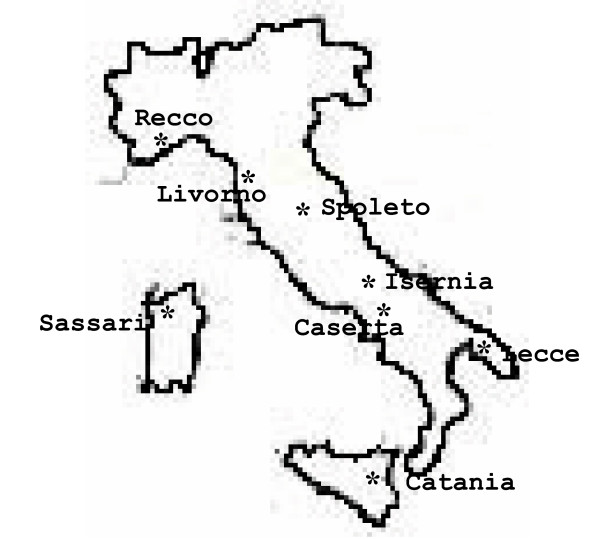
Collection sites of *A. acutifolius*.

Figure 1 and Table 1 show the eight different Italian sites where *A. acutifolius *was collected and their characteristics.

**Table 1:** Populations of *A. acutifolius*.

**Site**	**Abbr**	**Characteristics**					**N**
			
		**Hab**	**Alt**	**Exp**	**Ped**	**Assn**	
**Caserta Vecchia (Caserta)**	CAS	Clay hill	400 m	South, sunny	Acidic ground	Rubus	15
**Colli al Volturno (Isernia)**	ISE	Limestone hill	380 m	Semi-shadow	Alkaline ground	Quercus, Genista	16
**Lotrine (Livorno)**	LIV	Hedges	100 m	South-west, sunny	Alkaline ground	Quercus, Rubus	14
**Recco (Genova)**	REC	Torrent levee	5–10 m	West, shadow	Not recorded	Rubus	6
**Sassari (Sardinia)**	SAS	Coast	10–15 m	North, shadow	Neutral ground	Pinus	18
**Spoleto (Perugia)**	SPO	Cultivated fields	600 m	South- west, sunny	Alkaline ground	Quercus, Rubus, Genista	14
**Vignacastrisi (Lecce)**	LEC	Dry walls	50–100 m	North-west, shadow	Neutral ground	Quercus, Rubus	11
**Vizzini (Catania, Sicily)**	CAT	Ground cover	500 m	North, shadow	Neutral ground	Quercus, Rubus	15

Abbr, abbreviation; N, number of individuals; Hab, habitat; Alt, altitude; Exp, exposure; Ped, pedology; Assn, ecological association.

Among the 42 primers tested, 23 proved useful to characterize the samples (Table 2), whereas 19 were excluded due to absence of amplification (9 primers) or to amplification of the same single fragment in all samples (10 primers). The 23 useful primers gave a total of 228 polymorphic fragments, ranging from 150 to 1100 bp, with 100% repeatability. Fragments of the same molecular weight were considered as the same locus [17]. The validity of ISSRs in assessing genetic variability in the eight samples of Italian *A. acutifolius *is summarized in Table 3.

**Table 2:** ISSR primers useful for the amplification of the eight populations of *A. acutifolius*.

**Primer Name**	**Sequence 5'-3'**	**AT (°C)**
**3**	(CA)_8_AT	50
**4**	(CA)_8_AC	51.7
**5**	(CA)_8_GT	51.7
**8**	(CA)_8_GAC	54.7
**15**	GGTC(AC)_7_	56
**16**	CGTC(AC)_7_	56
**17**	CAGC(AC)_7_	56
**21**	CAGC(TC)_7_	56
**23**	GAG(TC)_8_	56
**8082**	(CT)_9_G	57
**8564**	(CAC)_7_T	58
**8565**	GT(CAC)_7_	58
**BEC**	(CA)_7_YC	54
**CHR**	(CA)_7_YG	54
**DAT**	(GA)_7_RC	54
**HAD**	CT(CCT)_3_CRC	54
**MAN**	CA(CCA)_3_CRC	54
**OH**	(GAG)_7_RG	66.7
**TE**	GT(GGT)_3_GRC	54
**W7**	(CT)_8_RG	52.8
**W814**	(CT)_8_TG	52
**W844**	(CT)_8_RC	52.8
**W902**	(GT)_6_AY	39

AT, annealing temperature.

**Table 3:** Genetic variability among the populations of *A. acutifolius*.

a)								
	**CAS**	**CAT**	**ISE**	**LEC**	**LIV**	**REC**	**SAS**	**SPO**
**3**	0.1554	0.2111	0.1018	0.1811	0.2157	0.0428	0.3172	0.0727
**4**	0.0599	0.3073	0.1664	0.1685	0.0000	0.0000	0.2753	0.1102
**5**	0.0581	0.1603	0.0823	0.1032	0.1059	0.1502	0.1037	0.0518
**8**	0.0698	0.1182	0.0667	0.1209	0.0621	0.0000	0.0563	0.1454
**15**	0.2086	0.2980	0.1330	0.2445	0.2612	0.1695	0.1734	0.1865
**16**	0.1661	0.1646	0.2129	0.1870	0.0470	0.1616	0.1820	0.1560
**17**	0.0846	0.1034	0.2370	0.0938	0.1660	0.2356	0.1616	0.1346
**21**	0.2490	0.2258	0.1345	0.1251	0.2058	0.1145	0.1185	0.1551
**23**	0.0711	0.1478	0.2607	0.0930	0.2663	0.0749	0.2746	0.2650
**8082**	0.1558	0.1163	0.1106	0.0127	0.0672	0.1474	0.1398	0.1395
**8564**	0.2033	0.0000	0.1453	0.2546	0.1567	0.0145	0.0825	0.1299
**8565**	0.2316	0.0924	0.1752	0.0783	0.1579	0.0856	0.1337	0.1001
**BEC**	0.0907	0.0860	0.1107	0.0475	0.1847	0.1471	0.1855	0.1250
**CHR**	0.2614	0.1785	0.0198	0.1980	0.0156	0.0530	0.0240	0.1161
**DAT**	0.1202	0.0676	0.1308	0.1706	0.1434	0.1699	0.1472	0.0905
**HAD**	0.1248	0.2192	0.1576	0.1725	0.1492	0.2171	0.2668	0.0529
**MAN**	0.2177	0.1119	0.2266	0.2135	0.0584	0.1394	0.0617	0.1455
**OH**	0.2527	0.2122	0.2099	0.2512	0.2150	0.1953	0.2615	0.1010
**TE**	0.0233	0.0559	0.0555	0.2077	0.0923	0.0525	0.1646	0.1333
**W7**	0.1613	0.1383	0.1502	0.1638	0.0738	0.1324	0.1987	0.1577
**W814**	0.1575	0.3503	0.2574	0.2106	0.0000	0.0000	0.3189	0.2179
**W844**	0.1063	0.2670	0.0962	0.0000	0.0000	0.2641	0.1253	0.0687
**W902**	0.2554	0.2413	0.2115	0.3393	0.1764	0.1636	0.1896	0.0895
**Mean ± SD**	0.1512 ± 0.1887	0.1539 ± 0.1940	0.1489 ± 0.1875	0.1625 ± 0.1964	0.1173 ± 0.1744	0.1180 ± 0.1771	0.1618 ± 0.1903	0.1259 ± 0.1842
**P**	50.44%	45.61%	46.49%	46.93%	39.91%	35.09%	51.75%	39.91%
**Number of specific bands**	3 M 9 P	4 P		4 P	2 P	2 P	3 P	1 M 3 P
b)								
	**H_T_**		**H_S_**		**D_ST_**	**F_ST_**	**Theta-B**	
**Mean ± SD**	0.2618 ± 0.0240	0.2859 ± 0.0155	0.1424 ± 0.0084	0.1619 ± 0.0026	0.1194	0.4561	0.4766 ± 0.0173	

a) Gene diversity for each primer set and population and over-all populations, and percentage of polymorphic loci (P) per population; the first column indicates the primer name; the last row indicates the number of population specific fragments (M = monomorphic, P = polymorphic);b) Mean values ± SD of total heterozygosity (H_T_), intrapopulation heterozygosity (H_S_) (Left column, POPGENE result; right column, Hickory result), diversity among population (D_ST_), fixation index (F_ST_), Theta-B.

A high level of genetic variation was observed using ISSR markers, with 100% polymorphic loci at the species level. The highest number of polymorphic loci (51.75%) was exhibited in the Sassari and the lowest (35.09%) in the Recco samples.

Genetic structuring was evident due to the detection of specific bands in each sample examined. Spoleto and Caserta samples showed one and three fixed specific fragments, respectively, found to be statistically significant (P < 0.0001). For the other samples, 27 ISSR specific polymorphic fragments were detected, with a varying degree of statistical significance ranging from P < 0.0400 to P < 0.0001.

Genetic distances [18] were examined for all pairwise comparisons between the sub-populations (Table 4). The mean distance for all comparisons was 0.1680, ranging from 0.0916 (between Isernia and Lecce) and 0.2865 (between Recco and Caserta). The Mantel test showed no correlation between the genetic and geographic data (-0.220).

**Table 4:** Genetic (below diagonal) and geographic (above diagonal) distances among the eight populations of *A. acutifolius*.

	**LIV**	**SPO**	**LEC**	**ISE**	**CAT**	**SAS**	**REC**	**CAS**
**LIV**	***	1.89	7.39	3.87	7.84	3.46	1.46	4.30
**SPO**	0.1354	***	5.53	2.12	6.53	4.19	3.27	2.65
**LEC**	0.1687	0.1134	***	3.58	4.17	8.13	8.80	3.33
**ISE**	0.1498	0.1257	0.0916	***	4.62	4.84	5.31	0.60
**CAT**	0.1180	0.1288	0.1321	0.1326	***	6.67	9.25	4.02
**SAS**	0.1265	0.1528	0.1404	0.1255	0.1096	***	4.12	4.86
**REC**	0.1388	0.1702	0.1821	0.1759	0.1267	0.1257	***	5.76
**CAS**	0.2591	0.2751	0.2591	0.2573	0.2421	0.2549	0.2865	***

**Figure 2:**
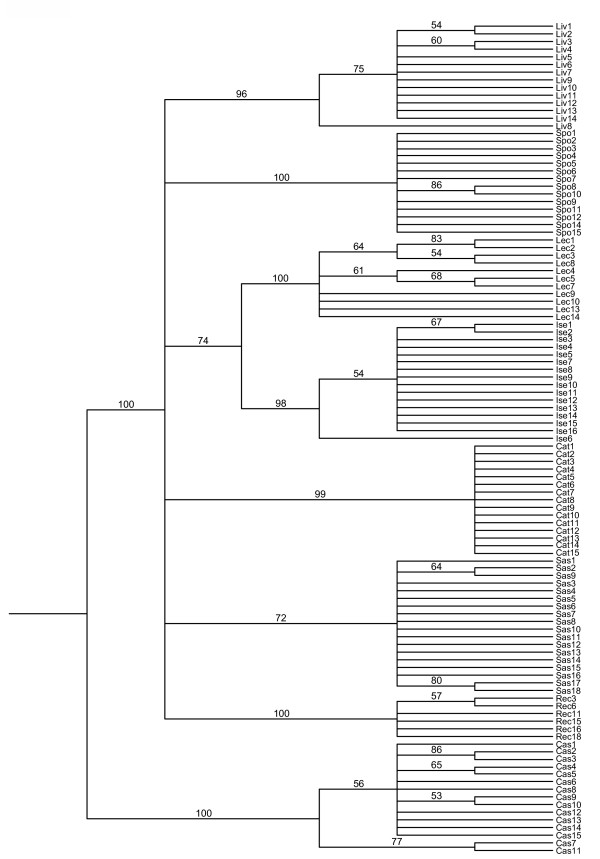
**UPGMA tree of the 109 *A. acutifolius *samples used in the ISSR analysis**. The numbers indicate the bootstrap values.

Samples collected at different geographic site grouped together, as shown in the UPGMA tree (Fig. 2), and the AMOVA analysis revealed significant genetic structuring (p = 0.001).

The values of gene diversity are summarized in Table 3a. For some primers, the value was 0.0000, and the highest value (0.3503) was found for the primer W814 in the Catania sample. This explains the high standard deviation values observed.

As summarized in Table 3b, the total variation (H_T_) was 0.2618 ± 0.0240 and the average variation within samples (average H_S_) was 0.1424 ± 0.0084. The mean diversity among the samples (D_ST_) was 0.1194. The fixation index F_ST _= (H_T_-H_S_)/H_T _was 0.4561, indicating a reduction of genetic diversity of about 45%. The Theta-B value obtained by Hickory analysis is an estimate of F_ST _under a random-effects model of population sampling. Its mean value is 0.4766 ± 0.0173; the H_T _and H_S _values are, respectively, 0.2859 ± 0.0115 and 0.1619 ± 0.0026 showing that there is a general agreement between the results obtained using the two different approaches.

## Discussion

ISSR markers can be used in population genetic studies of plant species as they effectively detect very low levels of genetic variation [19]. They also may have potential for analysing biogeographic patterns among populations of a single plant species. In this study, we have shown that these markers revealed genetic variation among geographically separated samples of *A. acutifolius *in an Italian population. ISSRs also revealed diversity within each sub-population. The results obtained are in accordance with the principle that the number of individuals used to estimate average heterozygosity can be very small if a large number of loci is studied [18].

The gene diversity values (Table 3a) ranged from 0.0525 (TE-Recco) to 0.3503 (W814-Catania). As expected, some primer-sample combinations showed no diversity (0.0) for two reasons: i) the combination primer-sample produced the same amplification pattern in all the samples (primer 4, Livorno and Recco; primer 8, Recco; primer W814, Livorno and Recco); ii) the combination primer-sample produced no measurable fragments (primer 8564, Catania; primer W844, Lecce and Livorno). These primers were not excluded from the analysis because in some cases they produced sample-specific fragments (e.g.: primer 8564 in Caserta and Lecce; W844 in Catania and Recco; primer 8 in Caserta, Catania, Isernia, Sassari, and Spoleto).

The fixation index is 0.4561, which indicates a substantial reduction of genetic diversity (about 45%), probably due to the high genetic isolation of samples analysed. The F_ST _and the Theta-B value (0.4766) demonstrated a very great genetic differentiation among samples, possibly caused by random genetic drift. Further statistical support to the genetic structuring of the samples examined comes from the AMOVA analysis. Despite the continuous distribution of *A. acutifolius*, the eight samples represent genetically differentiated populations.

In each population it was possible to identify ISSR specific fragments. As shown in Table 3a, the Caserta and Spoleto populations had specific fixed fragments that distinguished them from all others. Although the Isernia specific fragments are not statistically significant (P > 0.05), population-specific fragments were detected for all the other populations, with varying levels of intra-population polymorphism, ranging from 0.147 to 0.764. Thus, we have identified reproducible markers that distinguish the geographical origin of the *A. acutifolius *populations.

The high degree of genetic differentiation is confirmed by the UPGMA tree topology, in which all accessions from the same population grouped together (Fig. 2). The populations showed genetic distances ranging from 0.0916 and 0.1821 with the exception of the Caserta population that is more distant from the others (from 0.2421 with Catania to 0.2865 with Recco, Table 4). This result is probably due to the high number of specific ISSR fragments found in the Caserta population (12, including monomorphic and polymorphic) and can be attributed to genetic drift. In particular, the high distance between Caserta and Isernia (0.2573) is unexpected because of the short geographical distance separating the regions, and could explain the absence of correlation between genetic and geographical data matrices obtained with the Mantel test. The high genetic structuring of the eight populations shows that despite the continuous distribution of *A. acutifolius *throughout the Italian peninsula, there is poor gene flow through the isolates. The high genetic differentiation of the *A. acutifolius *populations examined might be attributed to the kind of pollinators (mainly bees) that can act at short distances, preventing the gene flow, and to the effects of anthropogenic habitat fragmentation.

The results obtained using ISSR markers are in agreement with the RAPD analysis that also identified population-specific fragments in different Italian *A. acutifolius *populations [12].

## Conclusion

Information about the spatial organization of genetic variability is essential for the conservation of genetic resources [20]. Our results provide an important contribution toward confirming that *A. acutifolius *has well-differentiated populations, despite their morphological low variability. These results show that to maintain genetic diversity within *A. acutifolius *it is necessary to conserve many populations.

## Methods

### Plant materials

A total of 109 samples of *A. acutifolius *collected in the eight different locations in Italy (listed in Table 1 and showed in Fig. 1) were used for the analysis. Although they are only a tiny fraction of the *A. acutifolius *Mediterranean distribution, they are representative of the Italian population.

### ISSR amplification

DNA was extracted from silica gel dried cladodes following the Doyle and Doyle protocol [21].

A total of 42 ISSR primers were tested on the eight populations of *A. acutifolius*. The polymerase chain reaction was conducted in a 9600 Perkin Elmer Thermal Cycler using the following reaction conditions: 2–5 ng DNA, 1.5 mM MgCl_2_, 0.2 mM dNTPs, 0.6 μM primer, 1.5 UE *Taq *polymerase (BIOLINE) and 1X *Taq *DNA polymerase buffer, in a total volume of 25 μL. The amplification programme was 1.5 min at 94°C; 35 × 40 s at 94°C, 45 s at the primer annealing optimal temperature (see Table 2), 1.5 min at 72°C; 45 s at 94°C, 45 s at the annealing temperature, 5 min at 72°C.

Following PCR, the samples were loaded onto a 1.5% agarose gel in TAE 1X buffer, stained with ethidium bromide. Additionally, 100 bp ladder (Promega) and negative and positive controls were loaded and run at constant voltage (150 V) for 2 hours. After running, the gels were UV visualised and recorded using a Kodak Digital Science dS1D DC40/DC120 Camera. To verify the repeatability of the results, each DNA extraction, PCR amplification, and gel running was repeated twice.

### Data analysis

Unequivocally scorable and consistently reproducible amplified DNA fragments were transformed into binary character matrix (1 = presence, 0 = absence).

Genetic variation within and among sub-populations was analysed on the basis of the banding profile using various parameters such as percentage polymorphism (P), total heterozygosity (H_T_), heterozygosity within population (H_S_), diversity among populations (D_ST_), fixation index (F_ST_), and genetic distance [18,22-24], using POPGENE software [25]. Since ISSR are dominant markers, data were also analysed using Hickory software [26] based on a Bayesian method that does not require prior decisions about the breeding system and Hardy-Weinberg equilibrium; the analysis was conducted under the f-free model.

AMOVA analysis, implemented in Arlequin [27], was conducted to document the degree of genetic structure among sub-populations.

The Mantel test of genetic and geographic distances was carried out to evaluate the correlation between the two data matrices.

The UPGMA tree was generated using the PAUP*4.0 software [28], and Bootstrap analysis was conducted using 1000 replicates.

## Authors' contributions

MS carried out part of the sample collection, designed ISSR primers and carried out ISSR work; GG did part of DNA extraction; SM carried out part of the sample collection and DNA extraction; LG participated in the manuscript preparation and revision; SA conceived the study, carried out data analysis, co-ordination and interpretation of the results.
